# A Wearable System for Jump Detection in Inline Figure Skating

**DOI:** 10.3390/s22041650

**Published:** 2022-02-20

**Authors:** Antonio Panfili, Alvise Spanò, Agostino Cortesi

**Affiliations:** Dipartimento di Scienze Ambientali, Informatica e Statistica, Università Ca’ Foscari Venezia, 30172 Venice, Italy

**Keywords:** wearable sensors, inline figure skating, sports biomechanics, human movement analysis

## Abstract

This article presents the design and experimental evaluation of a non-invasive wearable sensor system that can be used to acquire crucial information about athletes’ performance during inline figure skating training. By combining distance and time-of-flight sensors and gyroscopes, the system is able to detect when jumps are performed and provides a live view of the data (e.g., the number and height of jumps) through a graphical user interface. The main novelty of our approach lies in the way in which the optical sensors are orientated. Typically, the sensors are orientated horizontally and positioned in pairs on the ground, where they measure the time interval between the moment the athlete leaves the ground and the moment they land. In our system, an optical sensor is placed under each foot and is vertically orientated so as to constantly measure the distance from the ground. In addition, a gyroscope sensor is placed on the athlete’s back, which provides information on the direction and angular momentum of the movement. By combining this data, the system provides the accurate detection of various jumps and technical elements without any constraints on the training ground. In this paper, the system is also compared to similar platforms in the literature, although there are no other specific systems that are available for inline figure skating. The results of the experimental evaluation, which was performed by high profile athletes, confirm its effectiveness in correctly detecting jumps, especially considering its compromise between precision and the overall cost of the equipment.

## 1. Introduction

The availability of more precise sensors and more efficient data transmission technologies has allowed the development of systems that can be used to support the specialised technical preparation of athletes for different sports.

This work turns its attention to the sport of inline figure skating, for which sensor-based systems that are as sophisticated as those used in other sports disciplines have not yet been developed. Inline figure skaters train using roller skates, where the wheels are arranged on a single line and a piece of rubber at the front emulates the tip of the figure skating blade. In the future, our system could also be adapted for use in figure skating on ice and other winter sports as well [1].

### 1.1. Scenario

Firstly, we will provide some basic notions concerning equipment and athletic gestures, which we refer to as technical elements, or simply elements, from now on. To introduce the basic concepts relating to the technical elements, reference is made to a technical regulation, namely the ISU (International Skating Union) for the 2019/2020 competitive season [2] (for a comprehensive list of acronyms, see the table in the Appendix A). This choice was based on the quality and precision of how the elements are defined, as well as on the historical background of the ISU regulation that was approved during the 50th annual ISU congress in Scheveningen in the Netherlands, which is still in use today with minor changes carried out annually. Furthermore, the regulation adopted by the FIRS (Fédération International de Roller Sports), known as WS (World Skate) since the 2016/17 and 2017/18 competitive seasons, for inline figure skating is very similar to the ISU regulation. The final score of a competition programme is divided into the *technical element score* and the *programme component score*. The types of technical elements are: jumps, spins, step sequences and choreographic sequences. Figure skating jumps are grouped into different *families*: Salchow, toe loop, loop, flip, Lutz and Axel. Exceptions include: the *waltz* jump is only taken into account for youth categories because of its reduced rotation (half turn); the *Euler* jump (abbreviation: 1Eu), which is similar to a loop in that the landing foot has to be the opposite of the starting foot but is only considered when it is executed within a combination of jumps, i.e., between two jumps of the *families* listed above. The Euler, when used in combination between two listed jumps, becomes a listed jump (1Eu) with its value indicated in the SOV (scale of values) in the Technical Panel Handbook [3].

Notably, in the proposed sensor system, the type of jump is not taken into consideration; only the number of jumps and their height count. Each jump, excluding the *waltz*, must be performed with 1–4 rotations. Another exception is the *Axel*, for which jumps must be performed with $1\frac{1}{2}$–$4\frac{1}{2}$ rotations [3].

### 1.2. Challenge

The main challenge was to design a non-invasive wearable sensor system that could capture crucial information about an athlete’s performance during training. This information then needs to be processed to monitor and improve the relevant parameters of the training session. In particular, the following constraints were taken into consideration:

- the wearable device that is equipped with the sensors must not hinder the athlete or compromise their freedom of movement in any way;
- the system must not restrict the athletes to a limited area or to a specific surface with particular materials or lighting conditions;
- the number of athletes training simultaneously must not jeopardise the effectiveness of the system;
- the overall accuracy of the system determines the reliability of the product, hence the error correction applied to the raw data from the sensors is crucial;
- the cost is a relevant and decisive aspect for the choice of hardware of which the system is composed.

The detection of the technical elements is probably the most difficult task. It is not enough to detect whether an athlete has come off the ground and then measure the vertical distance: the real challenge is to filter out any vertical movements that would not be considered a valid jump or a technical element in a real performance.

### 1.3. Objectives

Our goal was to create a wearable device that is capable of accurately detecting, measuring and counting jumps performed by athletes when training for inline figure skating. The intended audience are, therefore, athletes during training sessions and not official competitions. We show that a system of non-invasive sensors can improve the effectiveness of training sessions by collecting data that are then analysed offline to provide further insights into performance.

As a minor objective, this information should be easy to access through a user-friendly graphic interface.

### 1.4. Contribution

The world of inline figure skating lacks the significant technological support that aims, above all, to provide athletes with useful information to improve their training sessions. Therefore, any automated feedback on the most relevant athletic elements is a valuable contribution to this field. From a scientific point of view, the main contribution of this study consists of carrying out the accurate measurement and detection of various jumps and technical elements by means of a single, non-invasive and inexpensive device that is equipped with sensors. No intervention to the training ground or environment is necessary.

The main novelty of this work is in the way that the optical sensors are orientated. Typically, sensors are orientated horizontally and placed in pairs on the ground to measure the time interval between the moment the athlete takes off from the ground and the moment they land again, as with those listed in Section 5.3. Our approach instead consists of installing an optical sensor under each foot and orientating it vertically in order to measure the foot’s distance from the ground constantly by sampling it several times per second. Additionally, one gyroscope–accelerometer pair is installed on the back of the athlete, providing a further source of information: namely, the direction and the angular momentum of motion. All data can then be combined and processed in real time to detect and count the jumps and to calculate the height and other parameters of the parabolic curve performed by the athlete.

### 1.5. System Architecture

**Figure 1:**
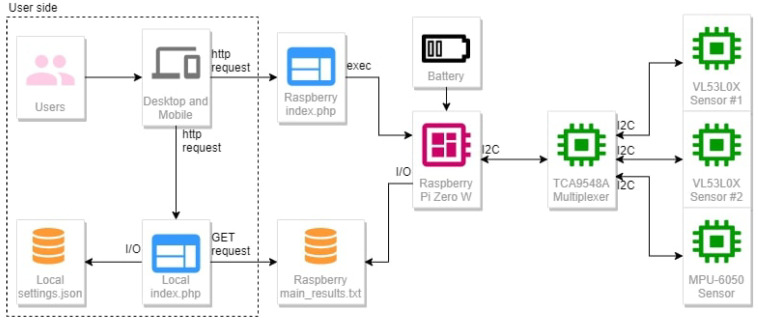
An overview of the system’s software and hardware components.

The overall architecture of our proposed system is depicted in Figure 1. It involves the use of distance and time-of-flight sensors and a gyroscope connected to a small computer that has the task of retrieving the sensors’ data and storing them for subsequent evaluation through a custom application, which is also discussed in this paper.

The system consists of two laser sensors placed under the skates (inside the frame) and a control unit placed on the back of the athlete, composed of an RPiZW board and a triaxial gyroscope. The two laser sensors and the gyroscope are connected to the control unit via cables passing through a multiplexer and the control unit is powered by a battery. When both the control unit and the user’s device are connected to the same network, it is possible for the user to start the sampling process using an easy and responsive GUI. When the sampling process is run, the control unit starts recording all sensors’ data into a shared text file in CSV format. At that point, the user can view a web page that automatically obtains all of the data from the shared file and, after the analysis, can show them all of the data either in real time (the updates run every 3 s) or in historic mode. Through this analysis web page, the user can edit some of the settings, such as the chart height, and add the IP address of the control unit.

## 2. Materials and Methods

In this section, we provide details of how we conducted our study and designed our system. In inline figure skating, one of the crucial factors is to optimise the jumps in any possible way, as they are the basic constituents of the technical elements. The ability to analyse the jumps in detail is a priority of our proposal: as shown in Figure 1, the two optical sensors installed under each skate produce enough data to draw two graphs, one for each foot, which show the athlete’s distance from the ground as a function of time, sampled about 10 times per second.

One important aspect of our system is that it does not attempt to infer the family of a jump—e.g., Lutz, Axel, Euler, etc. as listed in Section 1.1—but it detects any jump in the general sense by sampling the athlete’s distance from the ground. A jump is only detected when a given threshold is crossed several times in a row within a limited time interval. The algorithm and the detection strategy will be detailed in Section 2.4 and Section 3. Note that identifying the family of a jump was deemed as an unnecessary complication that, in the end, would not be worth the effort, as the athlete’s physical and biological parameters need to be taken into account to obtain precise results, such as their weight, height, dominant hand/foot, etc.

**Definition** **1** (Detachment)**.** *We considered the athlete as detached from the ground when the distance measured by both of the laser sensors between the skate and the ground surpassed a given threshold.*

This *threshold* was carefully calibrated through extensive testing and set by default to 20 cm. We calibrated this value by considering the distance between the sensor and the ground during the phase of the maximum inclination of the foot, including the front inclination, which was performed by climbing on both brakes by raising all three wheels, and the lateral inclination, which occurred during the execution of deep edges. This threshold did not depend on the athlete’s height nor on the size of their foot since the height of the frame was the same regardless of its length. However, this value could be modified through the user interface of the program in order to support changes in frame size, changes in wheel diameter, etc.

**Definition** **2** (Jump)**.** *We defined a jump as the contiguous series of N distance measurements performed by the laser sensors when they were detached from the ground, according to Definition 1.*

In this study, the value of N=3 was hard-coded into our algorithm as it yielded the best results according to our testing.

Our system was a compromise between accuracy and simplicity, being both lightweight from a computational point of view and easy to use from a user experience perspective. Most notably, it was not the actual distance measurements that mattered, but the trends, i.e., the derivatives, of the contiguous samples that were collected over time once the threshold had been reached.

### 2.1. System Requirements

Table 1 and Table 2 enlist the functional and non-functional requirements, respectively. The functional requirements were classified into user requirements (U), system requirements (S) and error recovery requirements (E). Since the system was divided into two components that interact with each other, i.e., the wearable device and the graphical display, we classified the non-functional requirements into general requirements (G), wearable control unit requirements (W) and user interface requirements (UI).

### 2.2. Hardware Components

The system comprised a wearable device that consisted of hardware components that respond to the following critical aspects. The size and weight of the wearable device must not affect athletic movement and must be comfortable for extended training sessions. The accuracy of sensors determines the reliability of the product as it impacts the quality of jump detection, which is the core feature of the system. Finally, the cost is the final important aspect as it affects the possible of uptake the system.

The hardware components that are depicted on the right-hand side of Figure 1 involved the use of the sensors and a single-board computer to perform readings, explain those readings and share them on a local network. The whole system was composed of:

- Raspberry Pi Zero W (https://www.raspberrypi.org/products/raspberry-pi-zero-w/ (accessed on 19 December 2021));
- TCA9548A multiplexer (https://www.ti.com/product/TCA9548A (accessed on 19 December 2021));
- Laser sensor STMicroelectronics VL54L0X (https://www.st.com/en/imaging-and-photonics-solutions/vl53l0x.html (accessed on 19 December 2021));
- MPU-6050 (https://www.radiolocman.com/datasheet/pdf.html?di=102365 (accessed on 19 December 2021));
- UPS-Lite v.1.2 (https://hackaday.io/project/173847-ups-lite (accessed on 19 December 2021)).

The Raspberry Pi Zero W was the single-board computer that we chose because it allows for communication with the sensors through the I2C protocol. The TCA9548A multiplexer was needed for addressing the sensors when retrieving data from them. The VL54L0X laser sensor was chosen for its small size and its accuracy in detecting distances, as well as for its eye safety as a Class-1 laser device that is compliant with the latest IEC 60825-1:2014 standard. The MPU-6050 was the gyroscope–accelerometer sensor that we used to filter the collected data. All sensors were connected to the multiplexer by means of cables and the whole system was powered by a polymer lithium battery through the UPS-Lite v.1.2 board.

### 2.3. Hardware Architecture and Sensor Placement

Ice Fly skates from EDEA srl (Crocetta del Montello (TV) Italy, https://ice.edeaskates.com/en/ice-skates/ice-fly/ (accessed on 19 December 2021)), a Snow White plate from Double L’s (http://www.inlinefigure.com/order_Snow_White.htm (accessed on 19 December 2021)), Zenith wheels (72 mm/78 A) from Roll Line T. M. Technology (Lancenigo (TV), Italy), D-stroyer Outdoor wheels (72 mm/81 A) from Kryptonics Wheels (El Segundo, CA, USA) and a Super professional ambra toe stop by Roll Line T. M. Technology (https://inlineartistic.roll-line.it/it/product/super-professional-ambra/ (accessed on 19 December 2021)) were used for the development of the project and for the tests.

**Figure 2:**
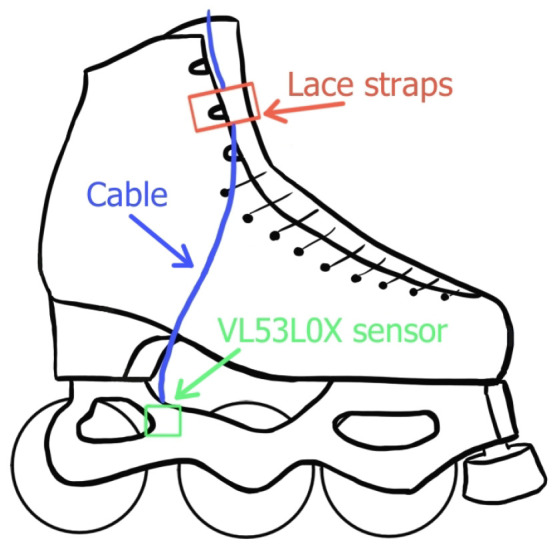
The positioning of the VL53L0X sensor on the boot.

The best position for the infrared sensors was between the second and third wheel, where the ground would be directly visible (see Figure 2). The minimum distance from the ground varied from 5 cm to 10 cm, depending on the type of skate installed under the boot, with its precise value being easily configurable through our software settings.

**Figure 3:**
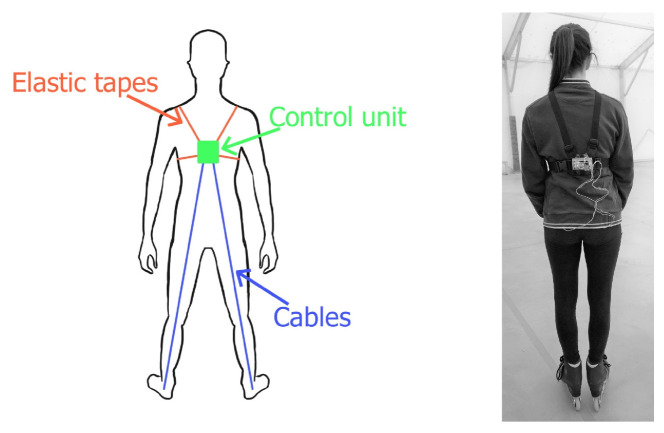
The positioning of the control unit on the athlete.

The RPiZW board, multiplexer, gyroscope–accelerometer and battery (control unit) were positioned on the mid-back of the athlete in a central position that would not be significantly affected by falls and would not unbalance the athlete (see Figure 3). In both ice skating and roller/inline skating, the majority of falls are in a forward direction. Skaters attempt to break their falls with their arms or hands in over 90% of falls in both ice skating (93.1%) and roller/inline skating (94.5%) [4]. The *control unit* was worn together with a simple harness made of a stretchy fabric, similar to those used for wearable action cameras.

The resulting system is depicted in Figure 1. The control unit consisted of the RPiZW board, powered by a battery. It communicated with the multiplexer via the I2C protocol through a cable connection, which managed the correct request addressing with the sensors. The I2C protocol provided easy communication without any data loss. It also provided an excellent speed compared to other protocols; I2C uses only two wires for communication so it is lightweight, economical and omnipresent. It also increased the data transfer rate [5]. Inside the control unit, there was also a MPU-6050 sensor that was connected to the multiplexer. The two VL53L0X sensors were connected to the multiplexer outside of the control unit.

### 2.4. Software Components

The data readings were managed entirely by the RPiZW board described in Section 2.2 (see Figure 1). The RPiZW represented the server of the I2C connection, with the sensors functioning as the client devices. It was, therefore, the RPiZW board that interacted with the sensors and stored the retrieved data in text files after processing them. The RPiZW board also hosted a PHP page: by accessing this page from a device connected to the same network, the user can start or stop the script that manages the communication between the RPiZW board and the sensors and saves results in TXT files.

The implementation was written in Python and was based on the *Adafruit* libraries that were released on GitHub under the name of *circuitpython* (https://github.com/adafruit/circuitpython (accessed on 19 December 2021)), which was a fork of the *micropython* project (https://github.com/micropython/micropython (accessed on 19 December 2021)). The Adafruit project allowed for communicate with sensors over the I2C bus. The Raspberry GPIO libraries (https://pypi.org/project/RPi.GPIO/ (accessed on 19 December 2021)) and Adafruit Blinka (https://pypi.org/project/adafruit-blinka/ (accessed on 19 December 2021)) were also used to communicate with the various pins of the RPiZW board.

Retrieving and storing data supplied by sensors was managed by a Python script executed by the RPiZW board. Its basic idea was similar to that proposed by [6]. The Python script read the values coming from the 3-axis accelerometer and 3-axis gyroscope within the motion sensor and stored them in CSV format. Algorithm 1 provided the implementation of this script in pseudocode. It established an I2C connection with the sensors, waited for their response and saved it in the */var/www/html* path. These files, therefore, contained the measurements for each sensor.
**Algorithm 1:** Sensor readings and sample storage.
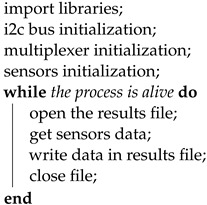


The data management was handled by an external device that was capable of executing PHP code. The task of this device was to execute a PHP page calling a JavaScript script every 3 s. The script executed a GET request through XMLHttpRequest() to retrieve the data from the created file (see Section 2.4) and used it to generate an evaluation chart. This PHP page used HTML code to set the content markup by using Bootstrap 4, JQuery (version: 3.4.1) and Plotly. Settings on the JSON file could be modified by the PHP page executing a POST. Notably, when the settings were changed, the UI triggered a repaint event and the charts were plotted from scratch.

Another important component was the code for generating the graphics of the page and the request for the script in JavaScript language. Algorithm 2 shows a pseudocode implementation of this. This script was responsible for executing the GET request to retrieve the TXT file generated by the Python script, which was located on the RPiZW board. It then analysed the data and displayed it on a chart using the **Plotly** library. The data analysis was performed considering the height of both feet from the ground. Once the limit threshold was exceeded, if the height of both feet remained above the threshold for at least three consecutive readings, then the series was highlighted by a red point on the graph and was considered a candidate for one single jump. The maximum height was always computed from the lower foot of the two when a candidate jump was detected for each foot.
**Algorithm 2:** Data processing and jump detection.
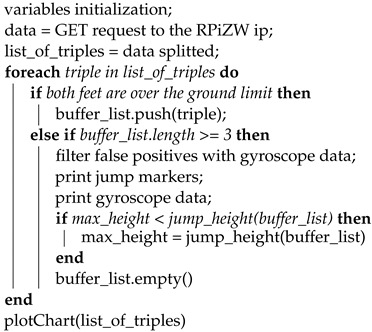


The execution speed of the jump detection script, implementing Algorithm 2, varied according to the number of sensors that were queried. By modifying the script, removing the interrogation part of the MPU6050 sensor and, thus, receiving only the values of the VL53l0X sensors, it was possible to estimate the difference in time taken to complete an overall cycle of data request, data acquisition and storage in a TXT file. The time estimation was performed by printing the timestamp of each iteration. On average, each cycle took about 93.63 milliseconds.

It is reasonable to claim that the system could provide at least 10 measurements per second. By similarly calculating the average time needed to complete a cycle to obtain the values of all three connected sensors, the elapsed time was just above 1/10 of a second with a difference of 8.13 milliseconds from the previous average. In quadruple jumps, the minimum flying time is 0.64 s (Salchow) and the longest flying time measured is 0.78 s (toe loop); therefore, skaters need more jumping power, especially to perform a quick take-off [7].

The average number of cycles per second of our system was, therefore, satisfactory since it allowed for the reading of at least three measurements during the flying phase, effectively satisfying functional requirement FR10 in Table 1.

### 2.5. Data Visualisation

**Figure 4:**
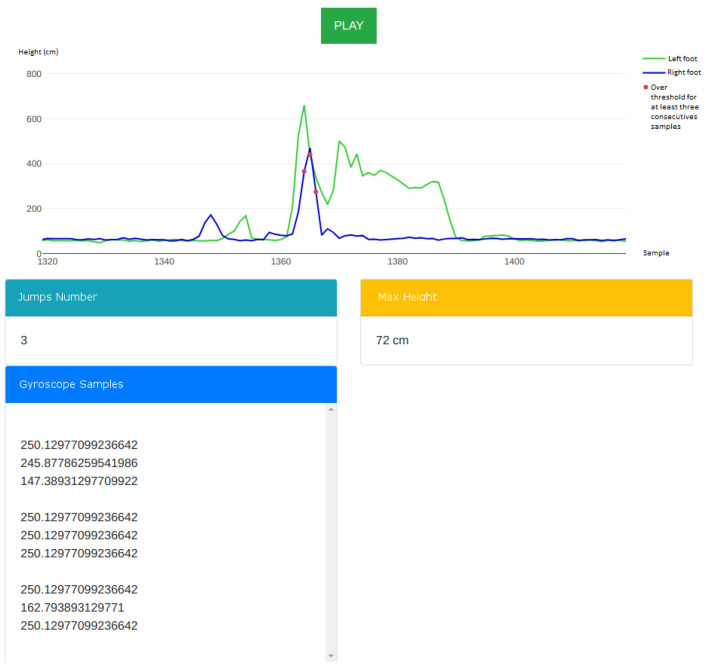
The web-based GUI. The graph shows jump heights sampled in real time by the sensors.

Figure 4 shows the web-based graphical user interface that was available to the user. A user could either be a computer operator aiding the athlete during training or the athlete themselves examining the data collected by the system at the end of the training session.

The screenshot shows the application when paused, where the following UI elements appear from top to bottom:

a [real-time graph] shows the jump heights, sampled by the two laser sensors;
the [“Number of Jumps”] displays the total number of jumps detected;
the [“Max Height”] displays the highest out of all detected jumps, with the lowest of the two feet always being reported;
the [“Gyroscope Samples”] displays the gyroscope values corresponding to the detected jumps.

When the application was in “play” mode, the user could monitor the data being collected in real time and see the graph being plotted with the jump heights sampled by the sensors. When in “pause” mode, the data retrieval stops temporarily, allowing the user to examine the data that was collected up to that moment, magnify the graph and inspect the values more carefully.

### 2.6. Experimentation on Human Subjects

We conducted multiple series of tests involving four humans subjects, two male and two female, who provided and signed an informed consent document. Two of them are high profile athletes in the discipline of inline figure skating, boasting a world-class career; the other two are athletes of regional and national level.

Each subject involved in the experiments was informed about the nature of the research and explicitly agreed for the research team to collect data while they performed jumps, technical elements or other athletic exercises.

All experiments were conducted in a gym under the supervision of a senior member of the research team and a senior athlete in the discipline of inline figure skating. The research presented in this paper and the experimentation involving human subjects were approved by the Ethical Committee of the Ca’ Foscari University of Venice.

#### 2.6.1. Experiment #1: Squat Jumps

For the first experiment, our subjects performed a series of 10 squat jumps each, with the purpose of evaluating the accuracy of the two laser sensors located under each foot. The squat jumps were executed wearing skates and from a standing position, using both feet to jump as high as possible.

As a reference for our measurements, we adopted an Abalakov belt [8,9]: a standard practice in sports involving jumps, which we discuss in detail in Section 5.3. It essentially consists of a rope bound to the athlete’s belt that is free to pull out during the vertical motion performed by the athlete. Once the athlete lands, the rope does not retract; hence, the amount of rope that pulled out equals the vertical distance covered during the jump.

#### 2.6.2. Experiment #2: Technical Element Detection

A second series of tests were conducted to evaluate the effectiveness of the technical element detection algorithm, which is the core feature of the proposed system. Our subjects performed a series of different jumps and technical elements on a regular inline figure skating rink (20 × 40 m${}^{2}$) with a quartz concrete surface.

As detailed in the Section 2.3, the materials used in our tests were:

**Skating boots:**  Ice Fly (athletes 1, 3 and 4) and Chorus (athlete 2) from EDEA srl;
**Frames:**  Snow White from Double L’s (all athletes);
**Wheels:**  Speedmax from Double L’s (athletes 1, 2, 3); Zenith (72 mm/78 A) from Roll Line T. M. Technology (second and third wheels) and D-stroyer Outdoor (72 mm/81 A) from Kryptonics Wheels (first wheel) (athlete 4);
**Toe stops:**  Ambra from Roll Line T. M. Technology (all athletes).

This equipment was provided by the research team in order to make tests as much consistent as possible.

## 3. Results

In this section, we report the results of our experiments and show the collected data and statistics.

### 3.1. Results of Experiment #1: Squat Jumps

Table 3 reports the following statistics of the squat jumps, conducted in a series of 10, for each athlete: the minimum, maximum and average heights $H_{min/max/\mu }$, read by the laser sensors; the average value $H_{\mu }^{ref}$ of heights, measured using the Abalakov belt; the minimum, maximum and average absolute deviation $D_{min/max/\mu }$ between the values read by the sensors and those measured by the belt; the variance $\sigma_{D}^{2}$ of the deviations, with respect to the average deviation $D_{\mu }$.

In this experiment, accuracy was defined as the ratio between the average absolute deviation $D_{\mu }$ and the average reference height $H_{\mu }^{ref}$:

$$\mathrm{Accuracy}=1-\frac{D_{\mu }}{H_{\mu }^{ref}}$$

**Figure 5:**
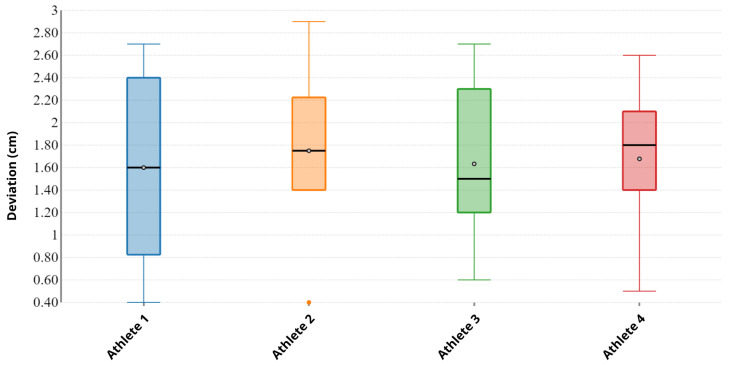
A box plot of the deviations between the sensor data and the belt reference measurement for each athlete. Empty dots represent the mean and black segments are the median. Despite the different physical characteristics of the athletes, the deviations were quite stable, with an overall mean of around 1.61 cm and a relatively low variance.

In Figure 5, we provide a box plot of the deviations between the values read by the laser sensors and those measured by the Abalakov belt, used as a reference, for each athlete.

### 3.2. Results of Experiment #2: Technical Element Detection

Algorithm 2 in Section 2.4 describes the technical element detection system as a three-stage pipeline implemented through a series of nested conditional computations. Table 4 shows the intermediate results for each of the three stages, testing the internals of Algorithm 2 on a series of different technical elements performed by the athlete subjects. Each column of the table represents a stage as a Boolean value.

**Has3Triggers:**  This indicates the first stage of detection; when the VL53L0X laser sensor detected three consecutive peaks of height above the threshold, a candidate jump was considered;
**IsCapped:**  When the Has3Triggers stage succeeded, up to five additional samples were taken into consideration for detecting the landing point of the candidate jump. Then, the values read by the MPU-6050 gyroscope were processed. When the values equalled the hard limit value 250 (refer to the appendix for the details of this hard limit), it meant the athlete was spinning too quickly and hence, that it was not a jump;
**IsJump:**  The rightmost column presents the ultimate result of a Boolean value indicating whether the given element was detected as a valid jump or not.

Table 5 reports the accuracy of the technical element detection at different thresholds for each athlete. These data involve non-vertical jumps while the athlete moved and performed complex spins and figures. The thresholds represent the minimum height at which our system considered a jump as a candidate technical element to be processed by the algorithm. The different physical characteristics and jumping capabilities exhibited by the athletes had an impact on the choice of threshold.

## 4. Discussion

In this section, we discuss our experiments and comment on the data collected therein and evaluate the effectiveness of the proposed system.

### 4.1. Discussion of Experiment #1: Squat Jumps

From data shown in Table 3, one relevant piece of information emerges: subjects of different gender, professional level, athletic performance and physical characteristics produced the same results in terms of accuracy, thus accuracy was not related to jump height. Secondly, Figure 5 highlights the overall low variance across the subjects, implying that the laser sensors were reliable in most scenarios.

### 4.2. Discussion of Experiment #2: Technical Element Detection

The data collected during the second experiment suggest that using N=3 as the number of contiguous samples above the threshold needed to trigger a valid jump (as in Definition 2) was a good trade-off. As a matter of fact, sampling heights 10 times per second allowed for three contiguous samples to cover a time span of 0.78 s, which is the average duration of a typical jump according to [7].

**Figure 6:**
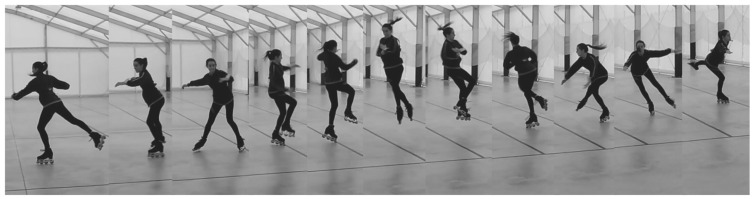
Double Salchow. Max height detected: 28.8 cm.

The first two technical elements, a double Salchow (see Figure 6) and a double toe loop, are essentially simple jumps and in both cases, our system always detected them with a threshold empirically calibrated to 20 cm. As the threshold increased, the jumps performed by athletes featuring the lowest average height were filtered out, i.e., not detected as valid elements. It is important to remark that three consecutive samples beyond the threshold had to be read by the software in order to consider it as a valid element. In some cases, the system did not detect a jump where the athlete surpassed the threshold for just 2 samples as a valid element.

**Figure 7:**
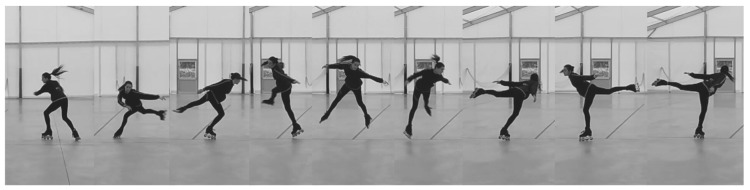
Flying camel spin. Max height detected: 23.6 cm. The element was discarded by the algorithm as it detected a fast spin after landing.

The flying camel spin (see Figure 7) is a spin with flying entrance, thus a jump takes place before starting the rotation on the floor. The system detected the jump entrance as an element, which was not supposed to happen and represented an error. Considering that the goal of a spin is not to achieve maximum height, the entrance jump reached a maximum height that was lower than that of an element based on a plain jump; hence, the number of errors decreased as the threshold increased. In order to avoid errors at lower threshold values, we introduced an additional filtering condition to the detection algorithm (see Algorithm 2 in Section 2.4).

Combination spins require one foot to remain on the floor. The obvious consequence of this was no incorrect detections arose with any threshold value because this element technically does not constitute a jump for our system, since both feet must pass the threshold for three samples for this to occur.

As far as a step sequence is concerned, the athlete has to perform difficult turns and steps and even choreographic elements, such as little hops or jumps. Only athlete #1 did not execute any jumps, even small ones, within the step sequence; hence, with thresholds higher than 15 cm, our system did not detect any jumps during the recording.

In all cases where the threshold was set to 10 cm, at least one element based on a jump was always incorrectly detected during the recording of the step sequence. The reason behind this was that the athletes might perform special movements where their feet slant with respect to the floor, causing the laser sensors to measure a further distance than the threshold due to the angle, especially when the threshold was set very low.

When setting the threshold at 20 cm, we achieved the best probability of success, which was increased even more by the algorithm filtering out false positives, such as spin jumps, through the control of the gyroscope values.

### 4.3. Accuracy of Jump Height Measurements

**Figure 8:**
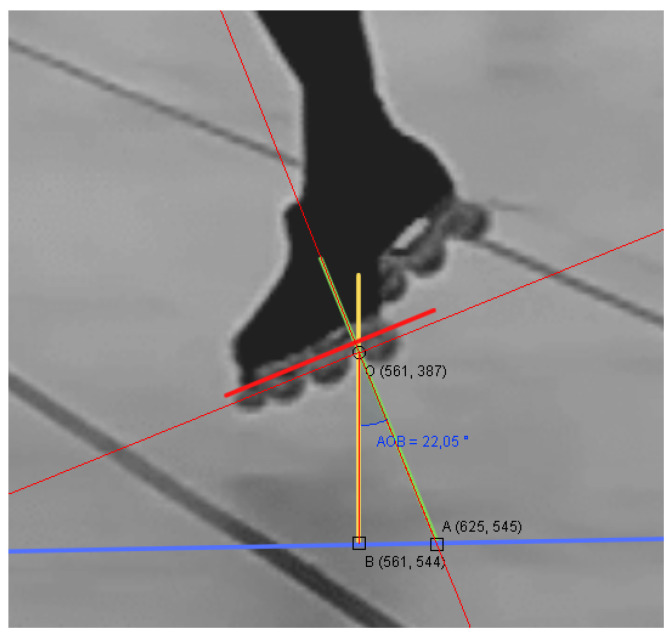
A sample calculation of vertical height $\bar{OB}$ given the tilt angle $\hat{AOB}$ and the distance from the ground $\bar{OA}$.

A mistake that an athlete can make when performing a technical element is to bend the legs or ankle during the jump; this caused the laser sensor installed under each skate to read a distance from the ground that was greater than the actual vertical height. Figure 8 shows an athlete bending legs in such a way that significantly impacted the measurements of vertical height due to tilting.

We analysed 40 videos (10 for each of the four subjects) that were recorded during our test sessions and, for each jump occurring in the performance, we extracted the angle between the foot and the ground by measuring it on the specific frame in which the highest tilt value appears, i.e., we picked the worst cases. In order to obtain an acceptable result, we only took into account frames where the athlete’s orientation was favourable for measuring such an angle, meaning that the third-dimension component was negligible.

With reference to Figure 8, we let $\bar{OA}$ be the distance from the ground as read by the sensor, $\bar{OB}$ be the vertical height and $\hat{AOB}$ be the tilt angle; then the equation $\bar{OB}=\bar{OA}\cos\left(\hat{AOB}\right)$ held.

Table 6 shows the vertical heights $\bar{OB}$ calculated for each combination of the minimum, maximum and average angles $\hat{AOB}$ and the minimum, maximum and average distances from the ground $\bar{OA}$, yielding nine results in total. The last column shows the accuracy, here defined as the ratio between the value $\bar{OA}$ measured by the laser sensor (the green hypotenuse of the triangle in Figure 6) and the actual vertical distance from the ground $\bar{OB}$ (the yellow vertical cathetus of the triangle in Figure 6). In other words, the accuracy was equal to the cosine $cos(\hat{AOB})$ of the tilt angle.

**Figure 9:**
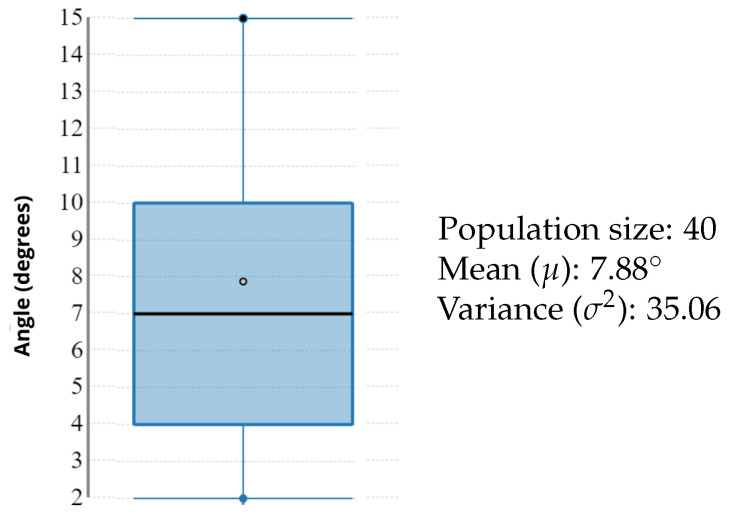
The statistics box plot of the 40 tilt angles measured from the video frames. The empty dot represents the mean and the black segment represents the median.

Figure 9 depicts the box plot of the tilt angle data extracted from the video recordings, displaying the relatively low variance and a mean of around $8^{\circ }$. As a general note, the system delivered a high accuracy overall: the mean angle ($8^{\circ }$) provided 99% accuracy; the upper bound of the third quartile ($10^{\circ }$) provided 98%; and the minimum angle ($2^{\circ }$) had an accuracy of 99%. Even the outliers that do not belong in the box plot in Figure 9 provided a good accuracy, such as the worst case of ${\hat{AOB}}_{out}=30^{\circ }$.

**Figure 10:**
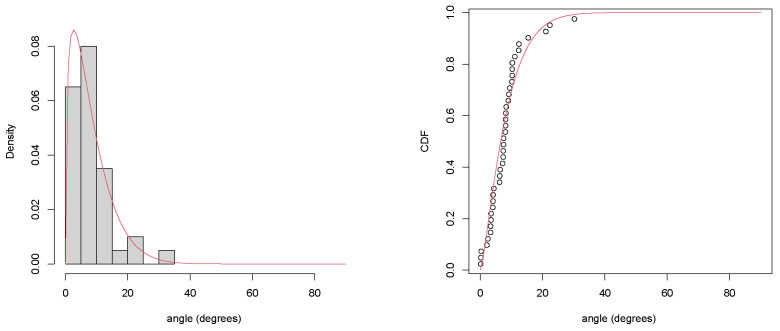
Inferential statistics on the population of tilt angles: the estimated density function in the range 0–90 degrees (**left**) and the cumulative distribution function (**right**).

In order to analyse the variability of the angles in our population, we modelled a beta distribution using the maximum likelihood method [10] for determining the α and β parameters. The domain of the beta distribution was rescaled into the $\left(0^{\circ },90^{\circ }\right)$ interval to match the whole possible range of the angular data, where $0^{\circ }$ represented the perfectly vertical orientation of the laser sensor with respect to the ground and $90^{\circ }$ represented the maximum absolute tilt. Figure 10 depicts the estimated density function and the cumulative distribution function (CDF) of this inferential model, showing, for instance, that an athlete’s foot exhibited a tilt angle lower than $20^{\circ }$ with roughly a 90% probability, hence an accuracy lower than $cos(20^{\circ })=0.93962\approx 94\%$ with a 10% probability.

### 4.4. In-Depth Analysis of a Jump

**Figure 11:**
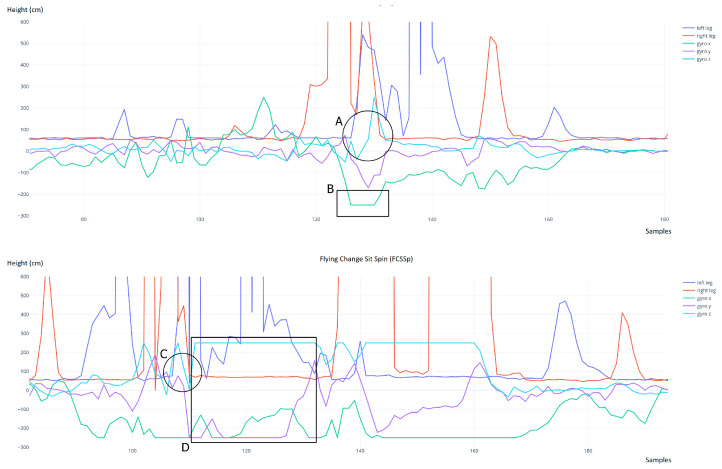
A comparison between a double Lutz (**upper chart**) and a flying sit spin with foot change (**lower chart**). The gyroscope hard limit was reached while jumping (the cyan line in the B area above) rather than after landing (the cyan line in the D area below).

We now delve into a detailed example of technical element detection consisting of a non-trivial jump. Figure 11 shows the data from the two laser sensors (blue and red) and the three axes of the gyroscope (green, purple, azure) during the execution of a double Lutz (upper chart) and a flying sit spin with foot change (lower chart). The hard limit value (250) reached by the various axes of the gyroscope (B) occurred when the athlete was in the air (A) during the rotation phase of the jump. Once landed, the gyroscope values dropped considerably. In the case of spins with a flying entrance, the data instead showed the persistent reaching of the hard limit of the gyroscope in each axis after the landing phase (D) and not just in correspondence with the flight phase (C).

It was, therefore, possible to reject false positives (flying spins) by performing an evaluation of the gyroscope data after the landing. The same differences were also observed in comparisons between the jumps and spins performed in the tests. Each test was performed three times, and all jumps were accurately detected with a properly tuned threshold value (20 cm). The harness did not disconnect during the tests and the device did not suffer any damage and remained bound to the harness without creating any difficulties in the execution of the elements.

**Figure 12:**
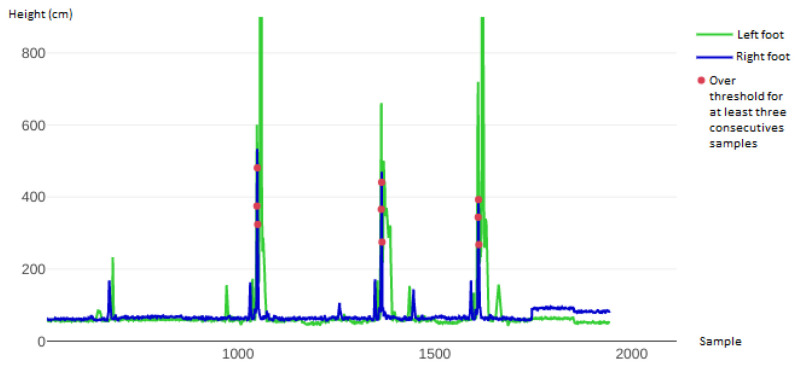
The green and blue lines relate to the left and right foot, respectively. The red dots highlight when both feet surpassed the threshold, with three red dots indicating that a valid jump was detected. This chart shows three successful detections of three technical elements (double Lutz).

Figure 12 depicts the chart related to three double Lutz jumps that were performed during the tests. The values read by the laser sensors mounted under the left and right skate are highlighted in green and blue, respectively. The red dots indicate that both feet exceeded the threshold: after three consecutive occurrences, the system identified the sequence as a valid jump. The graph in Figure 12 shows three groups of three red dots, meaning that all three double Lutz jumps were properly detected.

**Figure 13:**
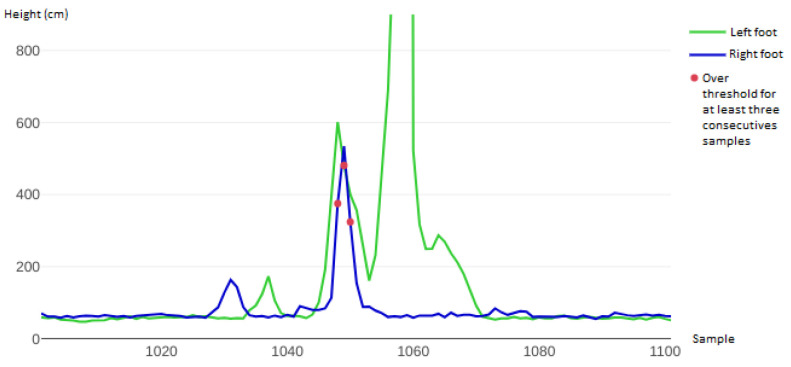
A zoom-in of the third (rightmost) double Lutz of the series of three in Figure 12. After the flying phase, marked by the three red dots and regularly detected as a valid jump, the left foot went parallel to the ground, hence the high green peak reaching the hard limit of the laser sensor, whereas the right foot (blue line) remained on the ground. This scenario corresponded to the exit phase of the element and was not detected as a jump.

Figure 13 zooms in on the rightmost part of the graph in Figure 12, showing the last double Lutz of the series of three in detail. The first peak below 200 mm (both in blue and green) represents the starting phase of the execution of the technical element. The following blue peak over 200 mm represents the take-off phase, the in air phase and the landing phase of the jump. At the same time, the green peak represents the left leg rising slightly more than the right as it helped the body in balancing its weight.

Finally, the landing phase was performed on the right foot only, since the blue line flattens much earlier compared to the green line. The green line then reached the hard limit of the sensor at 8190 mm, meaning that the left foot was so tilted that the sensor did not identify the floor: this represented the exit phase, where the left foot was indeed positioned parallel to the ground. However, since the right foot was on the ground, the system did not evaluate the exit phase as a jump, which was the correct behaviour.

## 5. Related Work

In this section, we discuss the most relevant papers in the literature that are related to our contribution. Distinguishing between cloud-based proposals and measurement systems, we compared our solution to a series of related tools that were, for the most part, not designed for inline skating. Almost no literature seems to exist on the discipline that this paper targeted, i.e., inline figure skating, although proposals worth a comparison do exist for similar sports, such as skating on ice, or for technologies based on similar principles.

### 5.1. Cloud Systems and BLE

Cloud systems, such as that proposed in [11], make data accessible everywhere, but also require a Wi-Fi connection, to enable the RPiZW board to access the Internet, or a GSM (Global System for Mobile Communications) module with a SIM (Subscriber Identity Module) to be added to the device. That is arguably a limitation in some scenarios. Our system does not depend on an Internet connection and can, therefore, be used anywhere.

A crucial aspect is the cost of time for uploading data from the wearable device to the processing device (a computer or a server). It requires processing time on the RPiWZ side and can, therefore, increase the time elapsed between contiguous sensor reads. A solution that does not need an Internet connection and uses BLE to transmit data from the sensors to a device, such as a smartphone, is presented in [12], where a mobile device manages the whole cloud upload process autonomously. Their system does not need a RPiZW board and resolves the constraints of a wired system, although it requires a BLE card, which increases the size of the two sensors (one for each foot). Our laser sensor is small enough to be placed in between the skate wheels, though adding a Bluetooth transmitter and a battery would make it impossible to fit.

### 5.2. Number of IMU Sensors

Several proposals in the literature use just one IMU sensor for each foot, for example in [13], whereas others take advantage of multiple IMU sensors.

In [14], up to 13 IMUs are adopted and the system is able to capture 6-degrees-of-freedom full-body motion in real time as long as there is at least one motionless foot at any given time. Generally, using multiple sensors placed in different key positions of the body allows for more precise measurements and more advanced results than simply detecting jumps and estimating their height. The ability to reproduce body movements in a 3D model would be more informational to the end user compared to our UI, but it would arguably rely on too many sensors, each powered by a battery and connected to a microcontroller that would use a BLE card to transmit the data to the server. Such a setup would be necessarily less lightweight and less comfortable than a system with fewer sensors, such as ours. Moreover, the IMU sensor that our system adopts is placed on the athlete’s back, both for safety reasons and because that placement is best for detecting body rotation [4].

The system proposed in [15], despite addressing ice skating rather than inline figure skating, is worth comparison due to method in which the measurements are carried out. Jump detection is based on waist-mounted IMUs and measures both the height and the rotation angle rather than only the height, as in our system. The height measurements take place at the peak of a jump, as with our algorithm, although the sampled values are subject to an error ranging between 3.3 cm and 7.81 cm, which is higher, on average, than ours. That paper also compares different algorithms for detecting valid vs. invalid jumps, whereas our system is based on one single algorithm tailored for yielding optimal results. Table 5 shows that the hit rate of our jump detection algorithm seems to be generally higher, assuming that a fine-tuned threshold is configured. The algorithms proposed in [15] depend on a few parameters that need to be manually configured; therefore, the paper does not provide a generic solution that adapts to all scenarios, whereas our system relies on one single parameter—the threshold.

### 5.3. Comparison to Other Systems

Many systems exist in the literature for measuring the different characteristics of a jump that are not related to skating as a sport. Most of these systems focus on providing the maximum height of the jump and collecting data for other purposes, although some provide a good amount of detail, such as the number of jumps performed, the intensity of the jumps and statistics related to a medical evaluation of athletic performance that is aimed at avoiding injuries.

- Sargent jump test [16,17];
- Abalakov jump test [8,9];
- Conductance platform [18,19];
- Insoles for pressure measurement: Pedar (https://www.novel.de/products/pedar/ (accessed on 19 December 2021));
- Optical detection system: Optojump (http://www.optojump.com/Cos-e-Optojump.aspx (accessed on 19 December 2021)) [18,19];
- Video triangulation system: TRACAB Optical (https://tracab.com/products/tracab-technologies/tracab-optical (accessed on 19 December 2021));
- Inertial measurement unit: XSENS (https://www.xsens.com/products/mvn-analyze (accessed on 19 December 2021)), VERT (https://www.myvert.com/ (accessed on 19 December 2021)) [20].

These systems feature useful aspects but none of them fully coincide with the design goals and requirements of our system, since they were not designed for the specific purpose of detecting jumps in figure skating. The Sargent jump test, Abalakov jump test, conductance platform and Optojump all require the jump to be performed within the measurement area, which is a major limitation. Despite Garcia-Lopez et al. [8] and Optojump offering a better overall precision compared to our system by measuring jumps through accurately detecting the flying curve performed by athletes, our system still detects technical elements with a high degree of reliability without imposing the above-mentioned space limitations and with a less expensive technology. On the other hand, the insoles for pressure measurement are not influenced by a measurement area though they present other issues, such as the size and weight of the instrumentation: the data analyser of the Pedar Insoles alone has a size of 15 × 10 × 4 cm and a weight of 400 g.

Analysing data from sensors is also required to determine when a jump, or another element, is occurring. Some products, such as the devices by the company VERT, are specialised in tracking the execution of jumps, while those by XSENS allow the reconstruction of the athlete’s movements and then animate a 3D model. Our proposal instead aims at detecting when a jump is performed, excluding any other elements performed by the athlete. Certain movements, such as flexing the knees and quickly extending them back to the previous position, could cause evaluation errors in systems that use IMU technology with few sensors for measurement. On the other hand, placing a lot of sensors in multiple key points of the body is not comfortable for the athlete and the cost of the resulting system would be pretty high, whereas our solution aims to reduce the number of sensors to the minimum whilst maintaining a high accuracy threshold and an affordable cost.

Out of the systems aimed at the competitive field, the best is arguably video triangulation. The major limitations of this technology, however, are the high cost of the equipment and the fact that it is highly conditioned by the position of the cameras, the type of background, the lighting of the environment and obstacles, including any other athletes in the same shot.

## 6. Conclusions

The system proposed in this paper is a hardware–software solution designed as a training aid for athletes in the discipline of inline figure skating. It is capable of interpreting data from non-invasive wearable sensors and detecting jumps and other elements executed by the athlete, ultimately providing a comprehensive view and analysis of their performance.

The sensors are installed under both skates worn on each foot and on the back of the athlete. The vertical distance measured by those sensors had an overall accuracy of 97% to 99% when the athlete does not bend their feet much while jumping, and even in the worst cases, an accuracy of over 85% was evaluated. The effectiveness of the jump detection system relies on these measurements and was proven to be 95% to almost 100% accurate, once a proper threshold was set. The threshold is a statically configurable value that represents the minimum distance from the floor that the system considers to be a jump and needs to be manually set by the user according to a number of physical parameters of the athlete, such as height, mass and strength. The experiments showed that a threshold of 20 cm yields good results for most athletes, despite their gender, skill and strength, as the results in Table 5 demonstrate. Furthermore, data from the gyroscope sensor is used to filter out false positives in cases such as flying spins, further increasing the overall quality of the detection.

If our results were to be confirmed by further tests conducted with a greater number of athletes and over a wider range of environments, locations, floor materials etc., that would arguably make the proposed system better than most jump detection systems available in the literature, especially those relying on video triangulation or on IMU wearable devices.

Among the major advantages of our system, it is capable of carrying out measurements with no space limitations and without depending on the strength of the Wi-Fi signal, which is usually an acceptable constraint in most cases as a Wi-Fi signal is easily extendable by installing signal repeaters that was readily available on the market. It also operates without a Wi-Fi connection once the acquisition script has been run, leaving data analysis for a later time.

The system has proven to be effective within training contexts where the counting and analysing of jumps and other technical elements is useful for a number of reasons, from improving overall athlete performance to fixing mistakes in execution.

### Future Challenges

Among the limitations of this system, its physical size is an open issue; a future upgrade could address this by crafting a custom case or box, whereas the current prototype was built by assembling consumer products that were already available on the market. We believe it is possible to greatly reduce the size of the device by carefully selecting finer (and more expensive) hardware. Removing the wires between the sensors and the RPiZW and adopting a fully wireless communication between the laser sensors and the RPiZW board would also be an important improvement as far as ergonomics are concerned. That would require a battery-powered communication board to be installed on the back of the athlete, rendering further miniaturisation all the more necessary.

## Figures and Tables

**Table 1:** The functional requirements.

	Functional Requirements	Type
FR1	The user must be able to start and stop the system from a user-friendly GUI.	U
FR2	The user must be able to view the data through a user-friendly GUI with a “real time” option.	U
FR3	The user must be able to delete the recorded data.	U
FR4	The user must be able to modify the system settings (e.g., server IP address, the minimum identification height of jumps, the vertical dimension of the graph, etc.).	U
FR5	The system must identify and display when a jump is performed.	S
FR6	The system must provide the user with the number of jumps identified since the beginning of the evaluation.	S
FR7	The system must provide the user with the maximum height reached in the jumps that were performed.	S
FR8	The system must provide correct data, even in the case of several athletes training on the same rink.	S
FR9	The system must not allow the user to modify the data obtained from the sensors.	S
FR10	The system must provide a minimum sample rate of eight times per second when reading data from the sensors.	S
FR11	In case of connection failure with the server IP address entered by the user, the system must continue to execute GET requests.	E

**Table 2:** The non-functional requirements.

	Non-functional Requirements	Type
NFR1	The athlete must be able to wear the device easily.	G
NFR2	The device must not hinder the athlete’s movements or affect them in any way (i.e., weight and position).	G
NFR3	The system must be usable in regular inline figure skating rinks (minimum 40 × 20 m).	G
NFR4	The control unit must run the Raspbian Lite or compatible operating systems.	W
NFR5	The control unit must communicate via I2C protocol with the sensors and obtain the necessary data with a frequency of less than or equal to three values per second.	W
NFR6	The control unit must grant permission to execute a script and terminate the process corresponding to the user interface.	W
NFR7	The control unit must grant permission to remove a specific file.	W
NFR8	The display of the graphical user interface must be independent of the type of computer used to run it and it must be responsive.	UI
NFR9	The user interface must communicate with the control unit through http requests.	UI
NFR10	The user interface must be run from any device capable of interpreting the PHP language.	UI
NFR11	The user interface must be viewable through a web browser.	UI
NFR12	The user interface must have permission to read and write files locally.	UI
NFR13	The user interface data update frequency must be greater than or equal to three times per second.	UI

**Table 3:** Squat jump measurements: the min/max/avg heights read from the sensors; the average reference height read from the Abalakov belt; the min/max/avg deviation between the data from the sensors and the belt; the variance of the deviations; accuracy.

Athlete #	$H_{\min}$ (cm)	$H_{\max}$ (cm)	$H_{\mu }$ (cm)	$H_{\mu }^{\mathrm{ref}}$ (cm)	$D_{\min}$ (cm)	$D_{\max}$ (cm)	$D_{\mu }$ (cm)	$\sigma_{D}^{2}$	Accuracy
1	20.3	23.6	22.11	23.71	0.4	2.7	1.60	0.73	93.25%
2	28.6	32.4	30.39	32.14	0.1	2.9	1.75	0.65	94.56%
3	25.3	29.6	27.70	26.07	0.6	2.7	1.63	0.51	93.74%
4	38.4	42.1	40.43	38.75	0.5	2.6	1.68	0.46	95.66%

**Table 4:** Intermediate Boolean states of the three-stage pipeline for the detection of technical elements implemented by Algorithm 2.

Element	Has3Triggers	IsCapped	IsJump
Push	No	No	No
Upright Spin	No	No	No
Sit Spin	No	No	No
Lunges Forward	No	No	No
Waltz Jump	Yes	No	Yes
Single Loop	Yes	No	Yes
Single Axel	Yes	No	Yes
Double Salchow	Yes	No	Yes
Double Toe Loop	Yes	No	Yes
Double Loop	Yes	No	Yes
Double Flip	Yes	No	Yes
Double Lutz	Yes	No	Yes
Flying Upright Spin	Yes	Yes	No
Flying Sit Spin	Yes	Yes	No
Flying Camel Spin	Yes	Yes	No
Flying Change Sit Spin	Yes	Yes	No

**Table 5:** The accuracy of the detection of different technical elements for multiple thresholds and for each athlete. The thresholds played a significant role in delivering optimal accuracy and were essentially dependent on the characteristics of each athlete, such as height, weight and strength.

Technical Element	Athlete #	Treshold						
		**10 cm**	**15 cm**	**18 cm**	**20 cm**	**25 cm**	**30 cm**	**35 cm**
Double Salchow		100%	100%	100%	100%	85%	50%	30%
	1	100%	100%	100%	100%	40%	0%	0%
	2	100%	100%	100%	100%	100%	60%	20%
	3	100%	100%	100%	100%	100%	40%	0%
	4	100%	100%	100%	100%	100%	100%	100%
Double Toe Loop		100%	100%	100%	100%	85%	50%	30%
	1	100%	100%	100%	100%	40%	0%	0%
	2	100%	100%	100%	100%	100%	60%	20%
	3	100%	100%	100%	100%	100%	40%	0%
	4	100%	100%	100%	100%	100%	100%	100%
Flying Camel Spin		0%	0%	12.5%	55%	100%	100%	100%
	1	0%	0%	40%	100%	100%	100%	100%
	2	0%	0%	0%	20%	100%	100%	100%
	3	0%	0%	10%	80%	100%	100%	100%
	4	0%	0%	0%	20%	100%	100%	100%
Combination Spin		100%	100%	100%	100%	100%	100%	100%
	1	100%	100%	100%	100%	100%	100%	100%
	2	100%	100%	100%	100%	100%	100%	100%
	3	100%	100%	100%	100%	100%	100%	100%
	4	100%	100%	100%	100%	100%	100%	100%
Step Sequence		0%	30%	95%	100%	100%	100%	100%
	1	0%	100%	100%	100%	100%	100%	100%
	2	0%	0%	100%	100%	100%	100%	100%
	3	0%	20%	100%	100%	100%	100%	100%
	4	0%	0%	80%	100%	100%	100%	100%

**Table 6:** The vertical heights $\bar{OB}$ calculated for each combination of the min/max/avg distances $\bar{OA}$ and angles $\hat{AOB}$.

	${\bar{\mathrm{OA}}}_{\min}=20.3$ cm	${\bar{\mathrm{OA}}}_{\max}=42.1$ cm	${\bar{\mathrm{OA}}}_{\mu }=30.2$ cm	Accuracy
${\hat{AOB}}_{min}=2^{\circ }$	$\bar{OB}=20.3$ cm	$\bar{OB}=42.1$ cm	$\bar{OB}=30.2$ cm	99%
${\hat{AOB}}_{\mu }=8^{\circ }$	$\bar{OB}=20.1$ cm	$\bar{OB}=41.7$ cm	$\bar{OB}=29.9$ cm	99%
${\hat{AOB}}_{max}=15^{\circ }$	$\bar{OB}=19.7$ cm	$\bar{OB}=40.8$ cm	$\bar{OB}=29.3$ cm	97%
${\hat{AOB}}_{out}=30^{\circ }$	$\bar{OB}=17.6$ cm	$\bar{OB}=36.5$ cm	$\bar{OB}=26.2$ cm	87%

## Data Availability

Data available in a publicly accessible repository that does not issue DOIs. Publicly available datasets were analyzed in this study. This data can be found here: https://github.com/alvisespano/sensors_2022_skate.

## Abbreviations

The following abbreviations are used in this manuscript:

AWG	American Wire Gauge
BLE	Bluetooth Low Energy
FDM	Fused Deposition Modelling
FIRS	Fédération International de Roller Sports
GND	Ground, Common Reference Potential of Value 0 V
GPIO	General Purpose Input/Output
I2C	Inter Integrated Circuit
IoT	Internet of Things
IIoT	Industrial Internet of Things
IMU	Inertial Measurement Unit
ISU	International Skating Union
JST-XH	Japan Solderless Terminal
PLA	Polylactic Acid
RPiZW	Raspberry Pi Zero W
SCL	Serial Clock
SDA	Serial Data
SOV	Scale of Values
VCC	Positive Power Supply
WS	World Skate

## Appendix A. The Hard Limit of Gyroscope Values

Each 16-bit gyroscope measurement has a full scale defined in FS_SEL (Register 27). For each full-scale setting, the gyroscope sensitivity per LSB in GYRO_xOUT is shown in the table below (Source: https://pdf1.alldatasheet.com/datasheet-pdf/view/1132807/TDK/MPU-6050.html (accessed on 19 December 2021)):

**Table A1:** Gyroscope sensitivity.

FS_SEL	Full Scale Range	LSB Sensitivity
0	±250°/s	131 LSB/°/s
1	±500°/s	65.5 LSB/°/s
2	±1000°/s	32.8 LSB/°/s
3	±2000°/s	16.4 LSB/°/s
